# Symptomatic Upper Extremity Deep Venous Thrombosis After Laser Lead Extraction

**DOI:** 10.21470/1678-9741-2022-0361

**Published:** 2023-07-14

**Authors:** Sameer Al-Maisary, Jamila Kremer, Gabrielle Romano, Matthias Karck, Raffaele De Simone

**Affiliations:** 1 Department of Cardiac Surgery, Heidelberg University Hospital, Heidelberg, Germany

**Keywords:** Subclavian Vein, Venous Thrombosis, Vascular Diseases, Arm, Morbidity

## Abstract

**Introduction:**

Laser lead extraction is a well-established method for removing unwanted leads with low morbidity and mortality. In this small series of cases, we documented our experience with venous thrombosis after laser lead extraction.

**Methods:**

Retrospective data of patients who underwent laser lead extraction with postoperative axillo-subclavian vein thrombosis between May 2010 and January 2020 were analyzed. Demographic, operative, clinical, and follow-up characteristics of those patients were collected from our medical database.

**Results:**

Six patients underwent percutaneous laser lead extraction. Mean age of the patients was 64±7 years. And four of them were male. A total of 11 leads with a mean age of 92±43.8 months were extracted. Patients presented with painful arm swelling postoperatively.

**Conclusion:**

Laser lead extraction may lead to symptomatic upper extremity deep venous occlusion.

## INTRODUCTION

**Table:** 

Abbreviations, Acronyms & Symbols	
AVB	= Atrioventricular block
CAD	= Coronary artery disease
CIED	= Cardiac implantable electronic devices
DM	= Diabetes mellitus
HTN	= Arterial hypertension
LBBB	= Left bundle branch block
LLE	= Laser lead extraction
LV	= Left ventricular
RA	= Right atrial
RV	= Right ventricular
SSS	= Sick sinus syndrome
VT	= Ventricular tachycardia

The implantation of cardiac implantable electronic devices (CIED) is a common procedure due to increasing indications for its use to improve survival and the quality of life in patients with cardiac rhythm disorders^[1,2]^. Proportional to this increase there is also an increase in the complications related to those devices, like infections and dysfunction, which makes its removal a necessity^[3-6]^. Many techniques were developed for lead extraction but most of them were ineffective especially in removing very old leads^[3,4,7]^. The introduction of laser lead extraction gave the doctors a good tool for lead extraction, which has a high success rate and a relatively low risk profile^[8-11]^. However, this low risk profile includes some serious complications that may alter the whole prognosis of a patient after laser lead extraction. Axillo-subclavian vein thrombosis is a rare illness. To the best of our knowledge, its association with laser lead extraction was not reported until now in the literature. The aim of this study is to turn the light on some of the cases in our cohort who had suffered from symptomatic deep vein thrombosis after laser lead extraction.

## METHODS

All patients who underwent laser lead extraction at Heidelberg University Hospital (Heidelberg, Germany), from May 2010 to July 2021, were retrospectively investigated. We screened the records to identify patients who suffered from axillo-subclavian thrombosis as postoperative complication of laser lead extraction. We gathered demographic, preoperative, intraoperative, and postoperative data of the affected subjects. The diagnosis of axillo-subclavian vein thrombosis was based on clinical symptoms, physical examination, and imaging tests.

Patients were referred from external hospitals or from our electrophysiological outpatient clinic. Indications for lead extraction were pocket infection, device-related endocarditis, pain, and abandoned or non-functioning leads at the time. Pocket infection was defined as redness with or without purulent discharge from the device pocket or device erosion which may be accompanied by pain. Device-related endocarditis was defined as persistent bacteremia or sepsis in the absence of another identifiable source or the presence of vegetations on the leads or valves. Pain related to CIED or leads was considered also as an indication for extraction. Extraction of abandoned or non-functioning leads is performed if they produce obstruction to a venous system or if a new lead implantation will increase the burden of the total lead number. The use of laser sheath was indicated if the removal of the leads under simple traction was not successful. All procedures were performed under general anesthesia with continuous arterial blood pressure monitoring. Under fluoroscopic guidance, the lead extraction starts by inserting a lead locking stylet into the inner coil lumen, then a suture is tied around the insulation and the locking stylet. After that, the laser sheath (GlideLight™ 80 Hz, 14 or 16 French) was advanced over the lead until the locking stylet emerged from the other side of the laser sheath. Laser was then applied while gradually advancing the sheath over the lead under traction until the lead was freed. Transesophageal echocardiography was used to monitor the procedure. In patients with local or systemic infection, no device would be implanted unless the patient is pacemaker dependent. In that case, an epicardial pacemaker lead is implanted through an inferior pericardiotomy^[12]^, and after remission of infection, the patient would be re-evaluated for dual-chamber pacemaker implantation.

For statistical analysis, continuous variables are expressed as mean or median and categorical variables are reported as frequency and percentages. Continuous variables were compared using a two-sample *t*-test.

The study proposal was approved by the Heidelberg University ethics commission (S-597/2019). All patients in this study consented to use of their medical records for research purposes.

## RESULTS

During the 11 years of study period, we performed percutaneous laser lead extractions in 274 patients, of which six patients (1.82%) suffered from axillo-subclavian vein thrombosis. Patient characteristics can be seen in Table 1.

**Table 1:** Demographic and clinical characteristics of patients with axillo-subclavian vein thrombosis after percutaneous laser lead extraction.

Patient No.	Age (years)/sex	Indication for CIED	Indication for LLE	Number of extracted leads	Age of leads (months)	Comorbid conditions
1	73/male	VT	Pocket infection	4	59	DM, HTN
2	68/male	VT and LBBB	Pocket infection	3	95	DM, HTN
3	66/male	VT	Lead perforation	1	146	HTN, CAD
4	62/male	AVB	Painful pocket	1	39	DM, HTN
5	53/female	SSS	Lead endocarditis	2	121	HTN, CAD
6	80/male	SSS	Pocket infection	2	100	CAD, HTN

AVB=atrioventricular block; CAD=coronary artery disease; CIED=cardiac implantable electronic devices; DM=diabetes mellitus; HTN=arterial hypertension; LBBB=left bundle branch block; LLE=laser lead extraction; VT=ventricular tachycardia; SSS=sick sinus syndrome

Mean age of the patients was 64±7 years. A total of 11 leads with a mean age of 92±43.8 months were extracted. All leads were completely extracted except in patient No. 1, in whom the double-coil lead was partially removed. Types of extracted leads are listed in Table 2.

**Table 2:** Types of extracted leads.

Patient No.	Single-coil lead	Double-coil lead	RV lead	RA lead	LV lead
1	1	1		1	1
2	1		1	1	
3		1			
4			1		
5			1	1	
6			1	1	

LV=left ventricular; RA=right atrial; RV=right ventricular

**Fig. 1:**
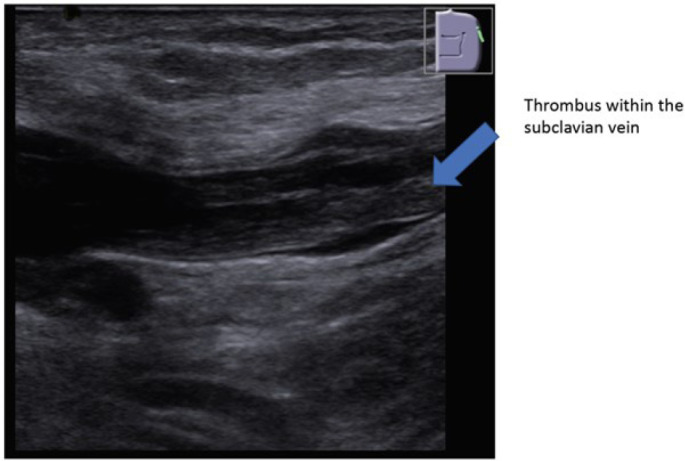
Duplex sonography showing thrombosis of the left subclavian vein.

**Fig. 2:**
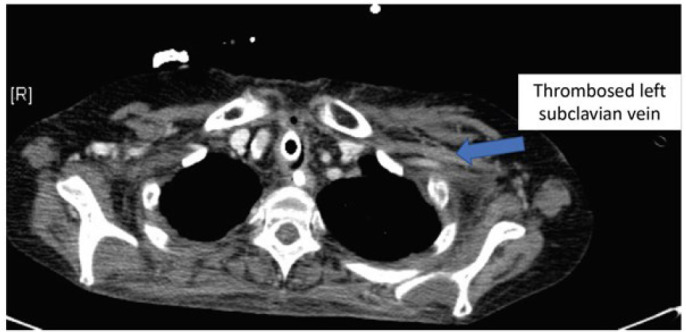
Computed tomography angiography showing thrombosed left subclavian vein.

All patients presented with arm swelling and purplish discoloration. Patients No. 1 to 4 had also pain in the affected arms. Patient No. 1, who received bilateral laser lead extraction, developed a superior vena cava syndrome with symptomatic bilateral arm swelling with pain and purplish discoloration and flushing. In patient No.1, computed tomography angiography confirmed the diagnosis and showed bilateral thrombosis of both axillary and subclavian veins and thrombosis of the innominate vein and the superior vena cava. In patients No. 2 and 3, a duplex ultrasound confirmed the thrombosis of axillary and subclavian veins on the side of laser lead extraction (Figure 1). In patient No. 3, the venogram showed preoperative stenosis of the left subclavian vein and postoperative thrombosis of the same subclavian vein. Patient No. 5 developed thrombosis of the subclavian vein, which was confirmed in computed tomography angiography (Figure 2). Patient No. 6 suffered from thrombosis of the left axillary vein after ipsilateral laser lead extraction, which was confirmed by duplex ultrasound.

**Fig. 3:**
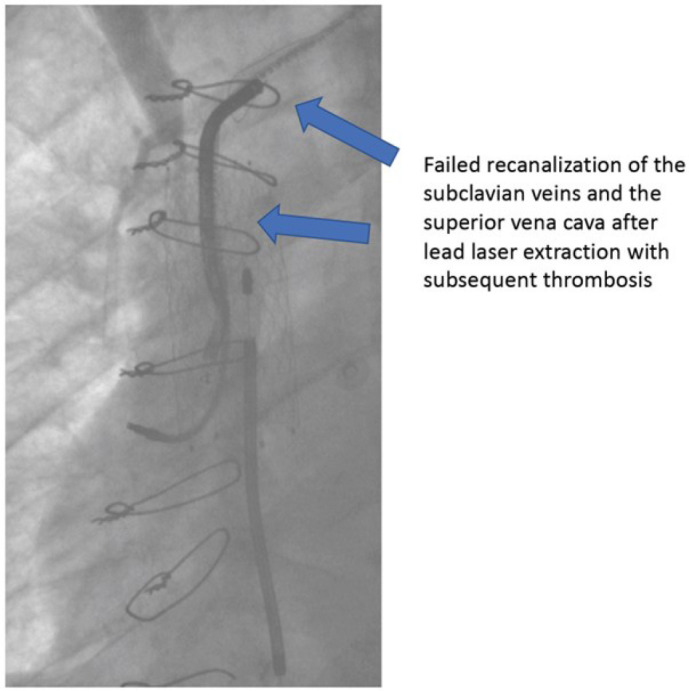
Venography showing rethrombosis of the superior vena cava after recanalization.

All patients received intravenous unfractionated heparin with elevation of the arm and compression bandage. Because of the severity of the symptoms, patient No. 1 received balloon angioplasty with stent implantation in the superior vena cava and the right subclavian vein. The symptoms diminished but two years later, a new phlebography showed complete rethrombosis of the superior vena cava and a balloon angioplasty was performed with initial success. However, five years later, complete thrombosis of the superior vena cava was detected, and no further intervention was undertaken (Figure 3). Patients No. 2, 3, 4, and 6 were treated medically and received anticoagulation for three months postoperatively. Patient No. 5 was also treated medically and due to worsening of his general condition, he was admitted to the intensive care unit. After developing pneumonia with respiratory failure, patient 5 died 87 days later.

## DISCUSSION

The pathogenesis of axillo-subclavian vein thrombosis after percutaneous laser lead extraction is not well understood. The long-lying leads develop adhesions and fibrosis in the venous system making simple removal troublesome. This fibrosis depends on the dwell time of the hardware and young patient age^[12]^. The use of laser sheaths bears the risk of inducing vessel or organ injury and sometimes embolism of tissue fragments^[8,11]^. As described by Tarakji et al.^[13]^, the removed leads contain remnants of vascular, atrial, and ventricular tissue, which creates abrasive surfaces in the venous system. The exposure of those surfaces to blood flow may trigger thrombus formation and vein occlusion. Albertini et al.^[14]^ has also detected a significant degree of upper extremity deep venous thrombosis occurring after laser lead extraction, which is often underdiagnosed. In addition, the remnant of partially removed leads, as in patient No.1, may also enhance this thrombotic effect. Also, the number of the extracted leads plays a role in this process as an increase in the number of laser rounds may increase the abraded surface area in the vein making thrombus formation more likely. The use of balloon angioplasty may alleviate the symptoms and gives time for the development of collateral circulation, but central vein patency may not be long-lasting. Generally, unilateral axillo-subclavian thrombosis can be well tolerated, and the symptoms subside over time under the use of anticoagulants with compression bandages and arm elevation, as collateral circulation develops to reduce the congestion. The only permanent problem is that the affected veins in our case series are not available for future lead implantation. Also, axillo-subclavian thrombosis may not cause complete venous occlusion, and in this way, it will be clinically symptomatic. This asymptomatic partial or complete occlusion of the axillo-subclavian vein will make future lead implantation questionable. Fu et al.^[15]^ reported three patients with superior vena cava syndrome after laser lead extraction with subsequent lead implantation. Also, Ghosh et al.^[16]^ reported three cases of venous thrombosis after laser lead extraction. This may raise the question, whether the venous thrombosis was a result of the extraction or the implantation^[17]^ or both. Also, the use of laser lead extraction in a stenosed vein, as in patient No. 3, may increase the chances of postoperative venous thrombosis as the generated tunnel is composed of injured surfaces, which are more amenable to thrombosis.

### Limitations

This study included a limited number of patients. A bigger multicentric study should give more information about such complications and the possible methods to manage them.

## CONCLUSION

Laser lead extraction may cause symptomatic venous occlusion affecting patients’ quality of life. A larger number of patients with a longer follow-up duration and frequent postoperative testing are needed to identify the patients at risk and to uncover silent venous thrombosis. Intravenous heparin followed by oral anticoagulants and venoplasty of the superior vena cava occlusion should help alleviating the symptoms. Discovering silent thrombosis may alter our approach to laser lead extraction.
